# Relationship between the sexual abuser and the victim

**DOI:** 10.1192/j.eurpsy.2021.1899

**Published:** 2021-08-13

**Authors:** L. Tumbev, P. Chumpalova, M. Stoimenova-Popova, V. Valtchev, E. Tumbeva

**Affiliations:** 1 Medical Psychology And Psychiatry, Medical University, Pleven, Bulgaria; 2 Department Of Biochemistry And Physiology, National Sports Academy, Sofia, Bulgaria; 3 Faculty Of Public Health, Department Of General Medicine, Forensic Medicine And Deontology, Medical University, Pleven, Bulgaria

**Keywords:** sexual crimes, victim, relationship

## Abstract

**Introduction:**

Although much of society believes that the sexual aggressor is unknown to the victim, this is not supported by the literature. In most cases, the rapist is a known, former or current intimate partner of the victim or even a family member.

**Objectives:**

189 persons accused of perpetrators of sexual crimes and who were subject to forensic psychiatric evaluation for the period from January 2010 to December 2019 in the territory of Central Northern Bulgaria were examined.

**Methods:**

The current research uses sociological methods to gather information - interviews, observations, research of forensic and medical documents,

**Results:**

In the study group, 62% of the victims were known to the perpetrator of the sexual crime, 11% were part of the nuclear family and 8% were members of the extended family of the perpetrator.

**Conclusions:**

Our data supports data from previous studies

**Disclosure:**

No significant relationships.

